# Effect of Sleeve Gastrectomy Versus One Anastomosis Gastric Bypass on Postoperative Renal Function and the Urinary Monocyte Chemoattractant Protein-1 (MCP-1) Level

**DOI:** 10.1007/s11695-023-07033-z

**Published:** 2024-01-09

**Authors:** Shimaa Monir Elmamlook, Alaa Abd El-Aziz Sabry, Mohamad Elrefai, Ahmed Bahie Eldeen

**Affiliations:** 1 Ministry of Health, Aga Central Hospital, Aga, Egypt; 2 Faculty of Medicine, Mansoura University, Jeehan Street, Mansoura, Dakahlia Egypt; 3 Gastrointestinal Surgical Center, Department of Surgery, Faculty of Medicine, Mansoura University, Mansoura, Egypt; 4 Clinical Sciences Department, Fakeeh College for Medical Sciences, Jeddah, Saudi Arabia

**Keywords:** Renal Function, Bariatric Surgery, Urinary MCP-1

## Abstract

**Introduction:**

Bariatric surgery has been the most effective treatment modality for morbid obesity that reduces associated comorbidities and improves quality of life. This study aims at evaluating and comparing the impact of two types of bariatric surgery—laparoscopic sleeve gastrectomy (LSG) and one anastomosis gastric bypass (OAGB)—on renal functions and urinary monocyte chemoattractant protein-1 (MPC-1) levels in morbidly obese patients 3 months after surgery.

**Methods:**

This is a prospective study of 40 morbidly obese patients who underwent bariatric surgery. Two types of bariatric surgery were done—laparoscopic sleeve gastrectomy (LSG) (26 patients) and laparoscopic one anastomosis gastric bypass (OAGB) (14 patients). The outcomes of the two procedures were compared in terms of renal function parameters and the level of urinary MCP-1.

**Results:**

There were no statistically significant differences in the mean postoperative urinary MCP-1 (73.53 ± 21.25, 75.43 ± 26.17, *P* > 0.5), microalbuminuria (8.83 ± 6.26, 10.02 ± 8.6, *P* > 0.05), urinary creatinine (109.21 ± 43.22, 99.19 ± 48.65, *P* > 0.05), MCP1/Cr ratio (0.78 ± 0.36, 1.01 ± 0.70, *P* > 0.05), eGFR (100.32 ± 9.54, 104.39 ± 9.54, *P* > 0.05) in the cases who had either LSG operation or OAGB operation.

**Conclusion:**

Bariatric surgery improves all indicators of kidney malfunction and reduces the level of urinary MCP-1. Both laparoscopic sleeve gastrectomy (LSG) and laparoscopic one anastomosis gastric bypass (OAGB) cause similar improvement of the renal function and reduction of urinary MCP-1 level.

**Graphical Abstract:**

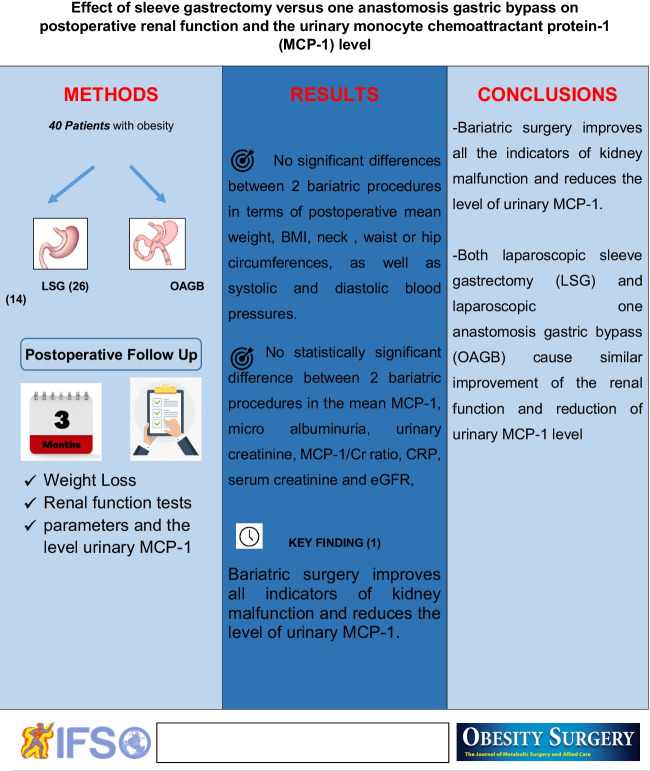

## Introduction

Bariatric surgery is considered the most effective treatment modality for morbid obesity resulting in significant improvement or reduction of comorbidities due to obesity [1].

A number of studies and meta-analyses have shown that albuminuria and proteinuria decrease after bariatric surgery, although it is unclear whether this is a direct effect of weight loss or mediated by improvements in blood pressure and insulin resistance. In the long term, bariatric surgery protects against renal function decline in patients with obesity [2–7].

Obesity has harmful effects on the kidney including obesity-related glomerulopathy (ORG), albuminuria, alternation of glomerular filtration rate, chronic kidney disease (CKD) and unfavorable outcomes in case of kidney transplantation [8]. Obesity-related glomerulopathy (ORG) is becoming a recognized cause of end-stage kidney disease. A slowly growing non nephrotic proteinuria is the most prevalent clinical manifestation, which is followed by a progressive decline in kidney function [9].

Monocyte chemoattractant protein-1 (MCP-1) (also known as CCL2) is a chemoattractant protein that belongs to a large family of chemotactic cytokines (chemokines) that are significant soluble mediators of innate immunity and tissue inflammation. MCP-1 was one of the earliest cytokines discovered to play a selective role in monocyte/macrophage migratory control [10]. MCP-1 is synthesized by a range of renal cell types, including intrinsic kidney cells and infiltrating leucocytes [11].

Post-bariatric reductions in MCP-1 are associated with improvements in renal function. Some of these changes are attributed to weight loss, as changes in the levels of MCP-1 that correlate with the degree of postoperative weight loss [2, 12].

## Patients and Methods

This is a single-center prospective study. In this study, 40 morbidly obese patients candidate for bariatric surgery were recruited. Two types of bariatric surgery were performed in this study: laparoscopic sleeve gastrectomy (LSG) and laparoscopic one anastomosis gastric bypass (OAGB). Twenty-six patients underwent LSG, and 14 patients underwent OAGB.

The study protocol followed guidelines of the 1975 Declaration of Helsinki, study protocol has been approved by the Institutional Research Board (Approval number: MS/17.05.64), and all included patients accepted participation in this work.

The choice of the operation between LSG and OAGB was based mainly on eating habits of the patient, long-term use of multivitamins, and follow-up visits (preferring OAGB for sweet eaters, preoperative gastroesophageal reflux (GERD), and comorbidities such as DM). However, patient’s choice was taken into consideration.

Inclusion criteria: both genders, age ≥ 18 to 60 years, mental competence, and morbid obese patient (BMI > 40 kg/m^2^). Exclusion criteria included age below 18 and above 60 years, chronic kidney disease (CKD) stage 3 or higher (eGFR < 60 ml/min), patients who were taking drugs which might interfere with glomerular filtration rate or decrease the level of protein in urine as inhibitors of the angiotensin-converting enzyme (ACE) or blockers of the angiotensin receptor (ARBs), patients with known renal disease not related to obesity such as systemic lupus erythromatosis and glomerulonephritis, patients with preoperative renal stones, patient unfit to surgery, revisional surgery (patients doing bariatric surgery for the second time), and patients unwilling to participate in the study.

Full history taking included patients, sex, age, associated co-morbidities, complete clinical examination, blood pressure, anthropometric and BMI measurements including waist circumference, hip circumference, waist-to-hip ratio and neck circumference, serum creatinine, estimated GFR, fasting blood sugar, lipid profile, serum albumin, C-reactive protein, urinary creatinine, albuminuria, and urinary monocyte chemoattractant protein-1 (MCP-1) were done to the patients before and after 3 months of the surgery.

Urinary MCP-1 level is measured using a commercial kit (catalog number CK-bio-12501) supplied by Shanghai Coon Koon Biotech Co, ELISA Kit.

### Statistical Analysis

The data was analyzed using IBM SPSS Corp.’s software, which was released in 2013. We used IBM SPSS Statistics for Windows. IBM Corporation (Version 22.0). Qualitative data was defined by number and percentage. Quantitative data was represented by median (minimum and maximum). Mean and standard deviation were used for parametric data. The significance of the obtained results was determined at the (0.05) level of significance.

## Results

This study included 40 patients with morbid obesity. The mean age of the cases was 35.55 ± 5.97 years with a range between 27 and 48 years, there was a significant increase in age of the LSG group than the OAGB group (36.96 ± 5.71 versus 32.93 ± 5.71 with *P* < 0.05). There were 11 males (27.5%) and 29 females (72.5%) in this study with 6 (23%) males in the LSG group versus 5 (35%) males in the OAGB group and 20 (77%) females in the LSG group versus 9 (65%) females in OMGB group) (Table 1).

**Table 1:** Age and gender and some laboratory data of 40 patients included in the study

Item	LSG group (*n* = 26)	OAGB group (*n* = 14)
Age (years)	36.96 ± 5.71	32.93 ± 5.71
Sex		
Male	6 (23%)	5 (35%)
Female	20 (77%)	9 (65%)
	All patients (*n* = 40)	
Fasting blood sugar (mg/dl)		
> 100	8 (20%)	
≤ 100	32 (80%)	
Blood pressure (mm Hg)		
≥ 130/80	6 (15%)	
< 130/80	34 (85%)	
TG level(mg/dl)		
< 150	26 (65%)	
≥ 150	14 (35%)	
HDL cholesterol level (mg/dl)		
≥ 45	19 (47.5%)	
< 45	21 (52.5%)	
LDL cholesterol level (mg/dl)		
< 130	24 (60%)	
≥ 130	16 (40%)	
Cholesterol level (mg/dl)		
< 200	17 (42.5%)	
≥ 200	23 (57.5%)	
Albuminuria(mg)		
< 30	20 (50%)	
≥ 30	20 (50%)	

Continuous data expressed as mean ± SD; categorical data expressed as number (%); *TGs*, triglycerides; *HDL*, high-density lipoprotein; *LDL*, low density lipoprotein; *mmhg*, millimeter mercury; *mg/dl*, milligram per deciliter

According to the preoperative data, 20% of the patients had impaired fasting glucose > 100 mg/dl, there were six cases (15%) with hypertension (HTN), 14 cases (35%) with triglyceride level ≥ 150 mg/dl, 21 cases (52.5%) with HDL cholesterol level < 45 mg/dl, 16 cases (40%) with LDL cholesterol level ≥ 130 mg/dl, and 23 cases (57.5%) with cholesterol level ≥ 200 mg/dl, half of the cases had more than 30 g urinary excretion of albumin.

Our results after surgery showed a statistically significant decrease in the mean postoperative weight, body mass index (BMI), neck, waist, and hip circumferences in both the LSG and OAGB groups in comparison with the preoperative values. There was a highly statistically significant decrease in the postoperative MCP-1, microalbuminuria, MCP-1/urinary creatinine ratio, and estimated GFR in both treatment groups in comparison with the preoperative value. Also, there was high statistically significant increase in the urinary creatinine and serum creatinine postoperative in comparison with the preoperative values (*P* < 0.001) (Table 2 and 3).

**Table 2:** Analysis of anthropometric measures, urinary-related parameters, and kidney function–related parameters before and 3 months after the operation in the LSG group

Item	Pre-operative (*n* = 26)	Postoperative (*n* = 26)	Test of significance
Weight (kg)	139.98 ± 20.13	116.92 ± 16.46	*P* < 0.001*
BMI (kg/m^2^)	50.57 ± 4.84	42.28 ± 4.25	*P* < 0.001*
Neck circumference (cm)	40.83 ± 2.34	36.83 ± 2.09	*P* < 0.001*
Waist circumference (cm)	136.85 ± 11.43	117 ± 11.15	*P* < 0.001*
Hip circumference (cm)	151.58 ± 10.30	133.46 ± 10.46	*P* < 0.001*
Waist/hip ratio	0.90 ± 0.04	0.88 ± 0.05	*P* < 0.001*
Urinary MCP-1(pg/ml)	103.95 ± 15.48	73.53 ± 21.25	*P* < 0.001*
Albuminuria (mg/l)	28.76 ± 36.06	8.83 ± 6.26	*P* = 0.004*
Urinary creatinine (mg/dl)	80.08 ± 55.14	109.21 ± 43.22	*P* = 0.002*
MCP-1/urinary creatinine ratio	1.97 ± 1.46	0.78 ± 0.36	*P* < 0.001*
Serum creatinine (mg/dl)	0.69 ± 0.04	0.74 ± 0.05	*P* < 0.001*
eGFR (ml/min/m^2^)	109.36 ± 15.93	100.32 ± 9.54	*P* < 0.001*

*P*, probability. Continuous data expressed as mean ± SD and (range); *statistically significant (*P* ≤ 0.05)

*kg*, kilogram; *BMI (kg/m*^*2*^*)*: body mass index (kilogram per meter square; *cm*, centimeter; *MCP-1*, monocyte chemoattractant protein-1; *pg/ml*, pictogram per milliliter; *mg/l*, milligram per liter; *mg/dl*, milligram per deciliter; *eGFR*, estimated glomerular filtration rate; *(ml/min/m*^*2*^*)*, milliliter per minute per meter square

**Table 3:** Analysis of anthropometric measures, urinary-related parameters, and kidney function–related parameters before and after 3 months of the operation in the OAGB group

Item	Pre-operative (*n* = 14)	Postoperative (*n* = 14)	Test of significance
Weight (kg)	151.57 ± 25.19	125.64 ± 19.83	*P* < 0.001*
BMI (kg/m^2^)	54.25 ± 6.06	44.60 ± 5	*P* < 0.001*
Neck circumference (cm)	42.21 ± 2.47	38.07 ± 2.21	*P* < 0.001*
Waist circumference (cm)	143.93 ± 12.80	123.71 ± 12.33	*P* < 0.001*
Hip circumference (cm)	155.57 ± 12.87	137.50 ± 13.49	*P* < 0.001*
Waist/hip ratio	0.92 ± 0.02	0.90 ± 0.02	*P* < 0.001*
Urinary MCP-1(pg/ml)	107.51 ± 15.10	75.43 ± 26.17	*P* < 0.001*
Albuminuria (mg/l)	50.40 ± 53.42	10.02 ± 8.62	*P* = 0.007*
Urinary creatinine (mg/dl)	70.29 ± 61.91	99.19 ± 48.65	*P* = 0.038*
MCP-1/urinary creatinine ratio	2.28 ± 1.38	1.01 ± 0.70	*P* = 0.001*
Serum creatinine (mg/dl)	0.71 ± 0.06	0.75 ± 0.07	*P* = 0.002*
eGFR (ml/min/m^2^)	114.49 ± 14.22	104.39 ± 9.54	*P* = 0.019*

*P*, probability. Continuous data expressed as mean ± SD and (range); *statistically significant (*P* ≤ 0.05)

*kg*, kilogram; *BMI (kg/m*^*2*^*)*, body mass index (kilogram per meter square; *cm*, centimeter; *MCP-1*, monocyte chemoattractant protein-1; *pg/ml*, pictogram per milliliter; *mg/l*, milligram per liter; *mg/dl*, milligram per deciliter; *eGFR*, estimated glomerular filtration rate; *(ml/min/m*^*2*^*)*, milliliter per minute per meter square

Additionally, there was a high statistically significant decrease in mean values of CRP, serum albumin, cholesterol, LDL, TGs, systolic blood pressure (SBP), and systolic blood pressure (DBP) 3 months after surgery in comparison with the preoperative values in both groups (*P* < 0.001).

Also, there was a high statistically significant increase in the serum level of HDL postoperative in comparison with the preoperative values (*P* < 0.001) in the LSG group. In the OAGB group, there was a high statistically significant decrease in the mean value of CRP, cholesterol, LDL, TGs, SBP, and DBP postoperative in comparison with the preoperative values (*P* < 0.001).

Also, there was a high statistically significant increase in the serum level of HDL postoperative in comparison with the preoperative values (*P* < 0.001) in the OAGB group (Table 4).

**Table 4:** Analysis of CRP, serum albumin, Lipid profile, and blood pressure–related parameters before and 3 months after the operation in the LSG group and OAGB group

Item	LSG group pre-operative (*n* = 26)	LSG group postoperative (*n* = 26)	Test of significance
CRP (mg/l)	7.14 ± 3.85	5.73 ± 3.04	P < 0.001*
Albumin (gm/dl)	4.48 ± 0.26	4.38 ± 0.26	P = 0.001*
Cholesterol (mg/dl)	206.69 ± 25.24	202.27 ± 24.31	P < 0.001*
LDL (mg/dl)	126.46 ± 18.24	123.27 ± 18.22	P < 0.001*
HDL (mg/dl)	47.27 ± 10.86	50.81 ± 10.42	P < 0.001*
TGs (mg/dl)	135.75 ± 35.11	112.15 ± 33.71	P < 0.001*
SBP (mmHg)	115.96 ± 12.73	109.81 ± 12.69	P < 0.001*
DBP (mmHg)	75 ± 7.75	70.77 ± 6.28	P < 0.001*

*P*, probability; continuous data expressed as mean ± SD; *t*, paired sample *t*-test; *statistically significant (*P* ≤ 0.05)

*CRP*, C reactive protein; *TGs*, triglycerides, *HDL*, high-density lipoprotein; *LDL*, low-density lipoprotein, *mmhg*, millimeter mercury; *mg/dl*, milligram per deciliter, *SBP*, systolic blood pressure; *DBP*, diastolic blood pressure; *mmhg*, millimeter mercury. *gm/l*, gram per liter; *mg/dl*, milligram per deciliter

Comparing the outcomes of both procedures, our results did not show any significant differences in terms of postoperative mean weight, BMI, neck, waist, or hip circumferences, as well as systolic and diastolic blood pressures.

Also, there was no highly statistically significant difference in the mean MCP-1, microalbuminuria, urinary creatinine, MCP-1/Cr ratio, CRP, serum creatinine, eGFR, cholesterol, HDL, and TGs in the cases who underwent either LSG operation or OAGB operation.

However, the mean serum level of albumin was statistically significantly higher in the patients who underwent LSG operation compared to the cases who underwent OAGB operation (Table 5).

**Table 5:** Analysis of studied postoperative parameters according to the type of operation

Item	LSG operation (*n* = 26)	OAGB operation (*n* = 14)	Test of significance
Post weight (kg)	116.92 ± 16.46	125.64 ± 19.83	*P* = 0.145
Post BMI (kg/m^2^)	42.28 ± 4.25	44.60 ± 5	*P* = 0.129
Post neck circumference (cm)	36.83 ± 2.09	38.07 ± 2.21	*P* = 0.086
Post waist circumference (cm)	117 ± 11.15	123.71 ± 12.33	*P* = 0.088
Post hip circumference (cm)	133.46 ± 10.46	137.50 ± 13.49	*P* = 0.301
Post waist/hip ratio	0.88 ± 0.05	0.90 ± 0.02	*P* = 0.084
Post SBP (mmHg)	109.81 ± 12.69	107.86 ± 8.02	*P* = 0.606
Post DBP (mmHg)	70.77 ± 6.28	70.71 ± 4.75	*P* = 0.977
Post urinary MCP-1 (pg/ml)	73.53 ± 21.25	75.43 ± 26.17	*P* = 0.788
Post albuminuria (mg/l)	8.83 ± 6.26	10.02 ± 8.62	*P* = 0.798
Post urinary creatinine (mg/dl)	109.21 ± 43.22	99.19 ± 48.65	*P* = 0.561
Post MCP-1/Cr ratio	0.78 ± 0.36	1.01 ± 0.70	*P* = 0.452
Post CRP (mg/l)	5.73 ± 3.04	5.77 ± 3.17	*P* = 0.971
Post serum albumin (gm/dl)	4.38 ± 0.26	3.99 ± 0.34	*P* < 0.001*
Post serum creatinine (mg/dl)	0.74 ± 0.05	0.75 ± 0.07	*P* = 0.648
Post eGFR (ml/min/m^2^)	100.32 ± 9.54	104.39 ± 9.54	*P* = 0.206

*P*, probability; continuous data expressed as mean ± SD; *t*, paired sample *t*-test; *statistically significant (*P* ≤ 0.05); *z*, Mann Whitney *U* test

*BMI (kg/m*^*2*^*)*, body mass index (kilogram per meter square; *SBP*, systolic blood pressure; *DBP*, diastolic blood pressure; *mmhg*, millimeter mercury; *MCP-1*,monocyte chemoattractant protein-1; *pg/ml*, pico gram per milliliter; *mg/l*, milligram per liter; *mg/dl*, milligram per deciliter; *Cr*, creatinine; *CRP*, C reactive protein; *eGFR*, estimated glomerular filtration rate; (ml/min/m^2^), milliliter per minute per meter square

There was a statistically significant positive correlation between preoperative MCP-1/urinary creatinine ratio with preoperative MCP-1. Also, there was a statistically significant negative correlation between preoperative MCP-1/urinary creatinine ratio with urinary creatinine (Table 6).

**Table 6:** Correlation between MCP-1/creatinine ratio (preoperative and postoperative) and other parameters

Parameter	MCP-1/creatinine ratio (preoperative)		MCP-1/creatinine ratio (postoperative)	
	Rho	*P* value	Rho	*P* value
Age (year)	− 0.159	0.328	0.008	0.962
Weight (kg)	0.031	0.851	− 0.208	0.199
BMI (kg/m^2^)	0.036	0.825	− 0.075	0.645
Neck circumference (cm)	− 0.031	0.847	− 0.208	0.198
Waist circumference (cm)	− 0.019	0.908	− 0.124	0.444
HIP circumference (cm)	− 0.123	0.449	− 0.169	0.296
Waist/hip ratio	0.110	0.499	− 0.017	0.915
Urinary MCP-1 (pg/ml)	0.496	0.001*	0.637	< 0.001*
Albuminuria (mg/l)	0.187	0.248	0.099	0.542
urinary creat. (mg/dl)	− 0.963	0.984	− 0.764	< 0.001*
CRP (mg/l)	− 0.003	0.886	− 0.186	0.249
Serum albumin (gm/dl)	0.023	< 0.001*	− 0.057	0.726
Serum creat. (mg/dl)	− 0.186	0.250	− 0.127	0.435
eGFR (ml/min/m^2^)	0.211	0.192	− 0.198	0.222
Cholesterol (mg/dl)	− 0.076	0.642	0.012	0.941
LDL (mg/dl)	− 0.002	0.990	− 0.040	0.809
HDL (mg/dl)	− 0.037	0.823	− 0.165	0.308
TGs (mg/dl)	0.055	0.736	− 0.165	0.308
SBP (mmHg)	0.046	0.776	− 0.106	0.514
DBP (mmHg)	0.055	0.735	0.008	0.962

Spearman’s correlation used; *highly significant *P* ≤ 0.001

*kg*, kilogram; *BMI*, body mass index; (*kg/m*^*2*^*)*, kilogram per meter square, *cm*, centimeter; *MCP-1*, monocyte chemoattractant protein-1; *eGFR*, estimated glomerular filtration rate; *pg/ml*, pico gram per milliliter; *mg/l*, milligram per litter; *mg/dl*, milligram per deciliter; *ml/min/m*^*2*^, milliliter per minute per meter square; *CRP*, C-reactive protein; *LDL*, low-density lipoprotein; *HDL*, high-density lipoprotein; *TGs*, triglycerides; *SBP*, systolic blood pressure; *DBP*, diastolic blood pressure; *mg/l*, milligram per litter; *mg/dl*, milligram per deciliter; *mmhg*, millimeter mercury

The results of our study demonstrated a significant positive correlation between preoperative glomerular filtration rate (GFR) with waist circumference (WC) and W/H ratio. There was a significant positive correlation between postoperative GFR with FBS, weight, WC, and waist/hip ratio postoperative (Table 7).

**Table 7:** Correlation between GFR (pre and postoperative) and some parameters

Variables	Preoperative GFR		Postoperative GFR	
	rho	*P* value	rho	*P* value
FBS (mg/dl)	0.168	0.300	0.322	0.043*
Weight (kg)	0.220	0.172	0.382	0.015*
Waist circumference (cm)	0.362	0.022*	0.414	0.008*
Waist/hip ratio	0.444	0.004*	0.370	0.019*

*r*, Spearman’s correlation; *p*, probability; *statistically significant (*P* < 0.05)

*FBS*, fasting blood sugar

As shown in Table 8, there was a statistically significant positive correlation between postoperative SBP with postoperative eGFR, weight, neck, waist, and hip circumferences. There was a statistically significant positive correlation between postoperative DBP with postoperative eGFR, weight, neck, waist, and hip circumferences.

**Table 8:** Correlation between SBP and DBP postoperative with other variables

Variables	Postoperative SBP		Postoperative DBP	
	rho	*P* value	rho	*P* value
Post eGFR (ml/min/m^2^)	0.372	0.018*	0.354	0.025*
Post weight (kg)	0.357	0.024*	0.403	0.010*
Post BMI (kg/m^2^)	0.080	0.624	0.256	0.110
Post neck circumference (cm)	0.411	0.009*	0.337	0.034*
Post waist circumference (cm)	0.424	0.006*	0.381	0.015*
Post hip circumference (cm)	0.406	0.009*	0.497	0.001*

*r*, Spearman’s correlation; *p*, probability; *statistically significant (*P* < 0.05)

## Discussion

Bariatric surgery has been the most effective treatment option for patients with morbid obesity resulting in significant weight loss, improved comorbidities, and improved quality of life [1].

In the morbidly obese population, weight loss that is attained through bariatric surgery results in an improvement in insulin resistance, oxidative stress, and inflammation [13, 14]. These improvements may contribute to the observed better outcomes after bariatric surgery in obese patients with CKD [15, 16].

A study done on 66 elderly obese patients showed that about 42% were diabetic, 50% were hypertensive, and 35% had hyperlipidemia [17].

The difference in the prevalence of hypertension and diabetes between our study and Nevo et al. [17] may be explained by two main factors: firstly, age difference as Nevo et al. performed their trial on elderly patients (mean age 67.6 ± 2.6 years) which was much higher than ours (mean age 35.55 ± 5.97 years). Secondly, in our study, patients who were on ARBs and ACEIs were excluded from this study due to their affection for glomerular filtration rate and proteinuria.

Results of our study demonstrated significant decreases in the mean postoperative weight, BMI, neck, waist, and hip circumferences in both LSG and OAGB groups in comparison with their preoperative values (Table 2).

The decrease in BMI in this study agrees with Alsharkawy and his colleagues, and BMI decreased significantly after 3 months postoperative. Body weight also had a significant reduction [18]. Said and his colleagues showed that at 6 months of follow-up, body weight and BMI showed significant improvement [19].

In this study, there was a highly statistically significant decrease in the postoperative urinary MCP-1, microalbuminuria, MCP-1/urinary creatinine ratio, and estimated GFR in both groups in comparison with their preoperative values. Also, there was high statistically significant increase in the urinary creatinine and serum creatinine postoperative in comparison with the preoperative values (*P* < 0.001) in all patients.

Our study results showed that renal function as evaluated by the eGFR improved 3 months after bariatric surgery in obese patients. By the estimation of GFR by modification of diet in renal disease (MDRD), mean eGFR decreased significantly. These results agree with a study of 57 patients with obesity, and MDRD-GFR was significantly decreased after 6 months of bariatric surgery [20]. Another study of 16 patients with severe obesity showed the median iohexol clearance rate was 109 [57–194] mL/min. The plasma iohexol clearance test showed hyperfiltration (mGFR > 120 mL/min) in seven patients [21]. In their study, the mean BMI (43.9 ± 7.3 kg/m^2^) was much lower than ours (51.85 ± 5.52 kg/m^2^); moreover, fewer number of their patients than ours may explain the difference. The discrepancy of these results could be explained by the difference in the preoperative filtration rate of the glomeruli. In patients with hyperfiltration, the glomerular filtration rate (either measured or estimated) decreased to the normal range; however, in patients with decreased GFR, resulting from the high prevalence of hypertension, diabetes, or chronic kidney disease, the GFR slightly increased as bariatric surgery improved hypertension and diabetes [22].

Regarding the decrease of CRP in our study, our results agree with a study which showed that serum CRP levels were significantly decreased at 12 months after surgery from (mean = 19.69 mg/l) to postoperative (mean = 16.11 mg/l) [23]. In another study, the serum CRP levels significantly decreased at 12 months after surgery from 25.7 ± 10.1 to 2.5 ± 0.7 mg/l with (*P* value < 0.01) [15].

Also, there was a high statistically significant increase in serum level of HDL postoperative in comparison with the preoperative values in both groups (*P* < 0.001) (Table 4).

Christiansen et al. [24] found that losing weight reduced MCP-1 concentrations, and their findings matched those of a meta-analysis published by Tannaz J et al. [25]. The mechanisms by which bariatric surgery improves endothelial damage biomarkers remain unknown. It is likely that adipose tissue loss is the primary mechanism responsible for the decrease in these markers [26]. 

The majority of pro-inflammatory cytokines began to diminish shortly after surgery and continued to decline in the medium and long periods. Tannaz J et al. [18] discovered that MCP-1 decreased with weight loss and that this decrease remained in long-term follow-up. Thus, metabolic improvement appears to be an early post-weight loss change that favors the resolution of obesity-induced inflammation [27].

There are important limitations of all observational studies of kidney disease and bariatric surgery, including potential residual confounding and the use of creatinine-based eGFR, which correlates with muscle mass. Loss of muscle mass with massive weight loss might result in overestimation of eGFR after bariatric surgery [28–30].

In patients with pre-surgical micro albuminuria, bariatric surgery can improve the urine albumin–creatinine ratio and cause the condition to remit [2, 31].

There are several ways that proteinuria can be reduced, but two main ones are better blood pressure and glucose homeostasis [5, 32, 33]. Nonetheless, there is a correlation between these enhancements and weight reduction, and it is plausible that the decreased inflammation linked to weight loss has a positive impact [2, 15].

We found that there were no statistically significant differences in the postoperative mean weight, BMI, weight, neck, waist and hip circumferences, waist/hip ratio, SBP, and DBP between the two bariatric procedures (LSG and OAGB). There were no statistically significant differences in the mean MCP-1, microalbuminuria, urinary creatinine, MCP-1/Cr ratio, CRP, serum creatinine, eGFR, cholesterol, TGs, and HDL in the patients who underwent either LSG or OAGB (Table 5). These results suggest that both types of bariatric surgery are equally effective in improving the postoperative outcome of the renal function and reducing the postoperative urinary MPC-1 level in morbidly obese patients.

However, the mean serum level of albumin was statistically significantly higher in the patients who underwent LSG operation compared to the cases who underwent OABG operation. This finding could be attributed to the malabsorptive nature of the bypass operation.

There was a statistically significant positive correlation between preoperative MCP-1/urinary creatinine ratio with preoperative MCP-1. Also, there was a statistically significant negative correlation between preoperative MCP-1/urinary creatinine ratio with urinary creatinine.

It is difficult to explain why there is no correlation between changes in BMI and the absolute difference in MCP-1 levels. BMI is not the best way to evaluate obesity, despite being the most commonly used indicator of obesity status in clinical and community health research. While BMI is an indirect indicator of obesity, it does not account for changes in body composition, distinguish between lean and fat mass (muscle mass, bone density, etc.), or consider where adipose tissue is located (visceral versus subcutaneous fat) [34].

There was a statistically significant positive correlation between GFR preoperative with WC and waist/hip ratio, and there was a statistically significant positive correlation between GFR postoperative with FBS, weight, waist circumference, and waist/hip ratio postoperative (Table 7).

As shown in Table 8, there was statistically significant positive correlation between postoperative SBP with postoperative eGFR, weight, neck circumference, waist circumference, and hip circumference. There was a statistically significant positive correlation between postoperative DBP with postoperative eGFR, weight, neck circumference, waist circumference, and hip circumference.

Limitations of this study are the relative few numbers of patients included with shorter period of follow-up (3 months).

## Conclusion

Bariatric surgery improves all indicators of kidney malfunction and reduces the level of urinary MCP-1. Both laparoscopic sleeve gastrectomy (LSG) and laparoscopic one anastomosis gastric bypass (OAGB) cause a similar improvement in the renal function and reduction of urinary MCP-1 level.

## Data Availability

Data generated or analyzed during this study are available from the corresponding author upon reasonable request.
